# CERCAM is a prognostic biomarker associated with immune infiltration of macrophage M2 polarization in head and neck squamous carcinoma

**DOI:** 10.1186/s12903-023-03421-0

**Published:** 2023-10-07

**Authors:** Ying Yang, Cong Yan, Xiao-Jian Chen

**Affiliations:** 1 Department of Stomatology, General Hospital of the Central Theater Command, Wuhan, 430070 China; 2 Department of Oral Maxillofacial-Head and Neck Surgery, School of Stomatology, China Medical University, Liaoning Provincial Key Laboratory of Oral Diseases, Shenyang, Liaoning 110000 P.R. China

**Keywords:** CERCAM, HNSCC, Prognosis, Macrophages, Immune infiltration

## Abstract

**Purpose:**

This study aimed to investigate the relevance of cerebral endothelial cell adhesion molecule (CERCAM) expression to head and neck squamous cell carcinoma (HNSCC) prognosis and immune infiltration by macrophage M2 polarization.

**Methods:**

Timer, UALCAN and HPA databases was used to analyze the differences in mRNA and protein levels of CERCAM expression in HNSCC. The Timer database was also applied to analyze the correlation between CERCAM in HNSCC and immune infiltration. TCGA-HNSCC database was applied to analyze the correlation between CERCAM expression levels and clinicopathological features, and its diagnostic and prognostic value in HNSCC was also assessed. The cBioPortal and MethSurv databases were then applied to analyze the genetic variation and methylation status of CERCAM. In vitro cellular assays were performed to provide evidence that CERCAM promotes malignant biological behavior of tumors and promotes macrophage M2 polarization in tumors. Finally, underlying pathophysiological mechanisms of CERCAM involvement in the development of HNSCC were predicted using a bioinformatics approach.

**Results:**

CERCAM is significantly overexpressed in HNSCC and correlates with poor prognostic levels and has good performance in predicting survival status in HNSCC patients. Cox regression analysis indicates that CERCAM expression levels are independent risk factors for predicting OS, DSS, and PFI. CERCAM promotes tumor malignant biological behavior and promotes macrophage M2 polarization immune infiltration in HNSCC. In addition, CERCAM promotes tumor cell adhesion in head and neck squamous carcinoma and promotes tumor progression through several oncogenic signaling pathways.

**Conclusion:**

CERCAM may serve as a new diagnostic and prognostic biomarker in HNSCC and is a promising therapeutic target for HNSCC.

**Supplementary Information:**

The online version contains supplementary material available at 10.1186/s12903-023-03421-0.

## Introduction

As the sixth most common cancer worldwide, head and neck squamous cell carcinoma(HNSCC) causes approximately 500,000 deaths and 400,000 new diagnoses each year [1]. Surgery is still the primary treatment for most head neck cancers, accompanied by other treatments such as radiotherapy, chemotherapy or immunotherapy [2–5]. Although great progress has been made in the diagnosis and treatment methods of HNSCC, the prognosis and survival of HNSCC have not improved significantly in the past decades, and the recurrence rate remains high and the 5-year survival rate remains low [6]. The pathogenesis of HNSCC has not been well explained so far. The abnormal expression of many genes in HNSCC may be involved in cancer development and progression [7–9], so searching for new gene markers involved in cancer development is currently the main direction of gene therapy.

Cell adhesion molecules are a collective term for the numerous classes of molecules that mediate contact and binding between cells and cells or between cells and the extracellular matrix (ECM) [10, 11].The adhesion molecules currently identified can be classified into integrin family, selectin family, immunoglobulin superfamily, calmodulin family according to their structural features [12–14]. In addition there are a number of adhesion molecules that have not yet been categorized. In tumors, tumor cells promote cancer progression through interactions with the cell or extracellular matrix to promote cell adhesion [15–17]. Cerebral endothelial cell adhesion molecule (CERCAM), a member of cell adhesion molecules, was originally identified in the blood–brain barrier [18], and many studies have shown that CERCAM is aberrantly expressed in many cancers and is also involved in cancer development and malignant progression [19, 20]. For instance, it has been shown that CERCAM is overexpressed in bladder cancer tissues and provided evidence in vitro that CERCAM promotes bladder cancer cell viability, DNA synthesis, and cell invasion, suggesting that CERCAM may function as an oncogene in bladder cancer [21]. And in our study, CERCAM was preliminarily found to be significantly expressed in HNSCC patients and associated with poor prognosis, suggesting that CERCAM may similarly play a role as an oncogene in HNSCC. Moreover, to date, the clinical significance of CERCAM in HNSCC and its biological relationship with HNSCC remain unclear, and few studies have been conducted. Therefore, this has attracted our attention and it is urgent to study this important topic.

The tumor microenvironment (TME) refers to the surrounding microenvironment that contains the presence of tumor cells, including the blood vessels surrounding the tumor, immune cells, fibroblasts, bone marrow-derived inflammatory cells, various signaling molecules, and extracellular matrix [22–27]. Studies have shown that tumor-associated macrophages (TAMs) in the tumor microenvironment can promote the malignant progression of many tumors [28, 29]. Macrophages can be classified into different biological properties as M1 macrophages, which is pro-inflammatory, and M2 macrophages, which is pro-immunosuppressive [30, 31]. Many studies have shown that M2 macrophages are involved in the progression of head and neck squamous carcinoma and are associated with their malignant biological functions [32, 33]. However, the role of CERCAM in influencing macrophage M2 polarization for cancer progression in HNSCC is still unclear.

In this study, we analyzed the expression pattern and function of CERCAM in HNSCC by bioinformatics methods and provided evidence in vitro that CERCAM promotes malignant biological behavior of tumors and induces macrophages M2 polarized immune infiltration to accelerate their malignant progression, in order to determine the clinical value and significance of CERCAM in HNSCC.

## Materials and methods

### Gene expression analysis

The Timer2.0 (http://timer.comp-genomics.org/) database was used to analyze the differences in mRNA expression levels of CERCAM in the pan-cancer range and their matched normal tissues [34]. The full name of the TCGA tumor abbreviation is given in Additional file 1 Table S1. The UALCAN database was used to analyze the difference in mRNA and total protein expression levels of CERCAM in HNSCC and their matched normal tissues [35]. Based on this database and analyzed for differences in mRNA expression in HPV-positive and HPV-negative and in TP53-mutated and TP53-nonmutated HNSCC patients. The GEO database dataset of head and neck cancer (GSE25099, GSE139869) was used to analyze the expression level of CERCAM in head and neck cancer. GSE25099 dataset was downloaded through the GEO database to obtain information on 22 normal samples and 57 tumor samples, and the information on CERCAM expression of these samples was statistically analyzed by t-test with normal and tumor groups, and a two-tailed P < 0.05 was considered to be statistically significant.GSE139869 dataset was downloaded through the GEO database to obtain information on tumor tissues and their corresponding paracancerous tissues of 5 HNSCC patients, and the CERCAM expression information of these samples was grouped by cancer and paracancer, and statistically analyzed using the paired t-test, and a two-tailed P < 0.05 was considered to be statistically significant. Immunohistochemical staining results from the HPA database of two patients were analyzed for differences in protein expression levels of CERCAM.

### TCGA data acquisition and analysis

Information on gene mRNA expression levels and clinical factors from 502 HNSCC patients was obtained in the TCGA database (https://portal.gdc.cancer.gov/) [36]. Patients were divided into CERCAM high expression group and CERCAM low expression group by the median of CERCAM expression levels to analyze the correlation between the difference in expression levels and clinical factors.

### Survival prognostic significance and diagnostic value analysis

Analysis of the relationship between CERCAM expression levels and overall survival (OS) and disease-specific survival (DSS) and progression-free interval (PFI) was performed, and hazard ratios (HR), 95% confidence intervals (CI) and *P* values were calculated. One-factor and multi-factor Cox regression analyses were performed to identify independent prognostic factors. Subsequently, to explore the prognostic impact of CERCAM expression in clinicopathological subgroups of patients with HNSCC, cox regression was used to analyze the relationship between CERCAM expression and OS, DSS, and PFI of each clinical subgroup of patients in TCGA-HNSCC, and the results were visualized by forest plot.

Based on the information from the HNSCC dataset in the TCGA database, ROC curves, time-dependent ROC curves, and column line graph models were developed through the Xiantao Academic Platform (www.xiantao.love) to visualize the diagnostic value of CERCAM expression levels in HNSCC patients.

### Genetic variation analysis

The cBioPortal platform (https://www.cbioportal.org/) was used to analyze the mutation frequency, mutation type, and mutation site information of CERCAM proteins in TCGA-HNSCC tumors as well as to visualize the 3D structure of mutant proteins [37].

### Methylation level analysis

We analyzed the differences in methylation levels of CERCAM genes between tumor tissues of HNSCC patients and their matched normal tissues by the UALCAN platform. The MethSurv platform was applied to analyze the DNA methylation levels in the CERCAM gene and the prognostic levels of CPG islands in the gene [38].

### Immune infiltration analysis

We used Timer1.0 (https://cistrome.shinyapps.io/timer/) database to analyze the correlation between the expression of CERCAM and the level of infiltration of six immune cells. In addition, this database system was also used to analyze the relationship between CERCAM gene expression levels and genetic markers of tumor infiltrating immune cells. And the correlation of CERCAM expression with M1 and M2 macrophage infiltration in patients with HNSCC, HPV( +) HNSCC, and HPV(-) HNSCC was studied by Timer 2.0 database based on two algorithms CIBERSOFT and CIBERSOFT-ABS.

### Single cell level analysis

TISCH2 (http://tisch.comp-genomics.org/home/) is a database for analysis at the single cell level that provides analysis of different functional states of cancer cells at the single cell level [39]. We used a HNSCC single-cell sequencing dataset (GSE103322) to analyze the correlation between CERCAM expression and HNSCC at the single-cell level.

### Gene co-expression analysis and enrichment analysis

The genes co-expressed with CERCAM in HNSCC were analyzed by LinkerOmics (http://www.linkedomics.org/login.php) database using Pearson correlation coefficients and the results were visualized by volcano plot and heat map [40]. The co-expressed genes were subjected to GO and KEGG enrichment analysis by online tool Xiantao Academic platform (www.xiantao.love) and the results were visualized [41–43]. The GSEA analysis module was also applied to visualize the enrichment results of CERCAM for differential genes in the high and low expression groups of HNSCC.

### PPI network establishment and hub gene analysis

We analyzed co-expressed gene information by String (https://cn.string-db.org/) online database, applied Cytoscape (v3.9.1) to extract the results of String database analysis, constructed PPI network, extracted significantly different modules by MCODE plug-in, and analyzed the modules by cytoHubba plug-in of hub genes.

### Cell lines and culture conditions

CAL-27 and SCC-9 were purchased from China center for type culture collection (Wuhan University, China), and THP-1 was purchased from Cell Bank, Chinese Academy of Sciences (Shanghai, China). CAL-27 and SCC-9 were cultured in DMEM (Gbico, USA) medium. THP-1 mononuclear cells were cultured in RPMI-1640 (Gbico, USA) medium with 0.05 mM β-mercaptoethanol. All cells were added with 10% fetal bovine serum(FBS), 1% penicillin/streptomycin and cultured in a cell culture incubator at 37 °C 5% CO_2_.

### Cell transfection assay

Small interfering RNA (siRNA) and negative control small interfering RNA (si-NC) were purchased from Ribobio Biologicals(Guangzhou, China). Their sequences are given in Additional file 1 Table S2. SCC-9, CAL-27 cells were cultured one day before transfection. The siRNA was first diluted in serum-free medium according to the manufacturer's procedure. Then INTERFERin® reagent was added to the siRNA-containing solution, vortexed and incubated for 10 min at room temperature. Finally, INTERFERin®-siRNA reagent was added to fresh complete medium and the cells were subsequently cultured. Transfection efficiency was detected by qRT-PCR for CERCAM expression to select the most suitable siRNA.

### RNA extraction and qRT-PCR

Quantitative real-time PCR (qRT-PCR) was performed to assess mRNA expression levels of M2 macrophage-related genes: CD163, VSIG4, CD206. Total RNA was extracted from cultured cells and reverse transcribed into cDNA using Trizol reagent (TaKaRa, Kyoto, Japan) and PrimeScript RT kit (TaKaRa, Kyoto, Japan) according to the manufacturer's recommended protocol. Subsequently, one-fifth of the cDNA was used as template for qRT-PCR using the TB Green® Premix Ex Taq™ II kit (TaKaRa, Kyoto, Japan) via an ABI QuantStudio3 Real-Time PCR system (Applied Biosystems, Foster City, CA). Relative gene expression was calculated using the 2^−ΔΔCT^ method. The housekeeping gene GAPDH was used as an internal standard control. Primer sequences designed by Sangon Biotech (Shanghai, China) were given in Additional file 1 Table S3.

### CCK-8 proliferation assay

The transfected HNSCC cells were cultured in 96-well plates at a density of 5000 cells per well in 100 μl medium per well. After 24 h, 48 h, 72 h, and 96 h, the supernatants were replaced with 100 μl of fresh medium containing 10 μl of CCK-8 solution (Beyotime, Shanghai, China), respectively. The cells were incubated for an additional 1 h at 37 °C in the dark. Subsequently, the optical density (OD) value of each well at a wavelength of 450 nm was measured using an enzyme marker (Tecan infinite M200).

### Cell adhesion assay (MTT)

The 96-well plate was spread with 50 μl of fibronectin adhesive solution (Beyotime, Shanghai, China) at a concentration of 10 μg/ml per well, and air-dried overnight in an ultra-clean bench. On the second day, the coating solution was discarded, and 200 μl of 1% BSA was added to incubate the well plates at 37℃ for 1 h, and then the well plates were washed three times with serum-free medium to wash away the excess gel solution. The transfected HNSCC cells were inoculated in 96-well plates at a density of 50,000 cells per well in the experimental group and the control group with serum-free medium in a volume of 100 μl per well. The cells were incubated in a cell culture incubator for 60 min, and then the original medium was replaced with 100 μl of fresh medium containing 10 μl MTT solution (Beyotime, Shanghai, China, 5 mg/ml). The transfected HNSCC cells were inoculated in 96-well plates at a density of 50,000 cells per well in the experimental and control groups using serum-free medium in a volume of 100 μl per well. The cells were incubated in a cell culture incubator for 60 min, and then the original medium was replaced with 100 μl of fresh culture medium containing 10 μl of MTT solution (Beyotime, Shanghai, China, 5 mg/ml) and incubated at 37 degrees Celsius for 4 h. After 4 h, the supernatant in the wells was aspirated and 150 μl of DMSO was added to each well, and shaking bed was shaken for 15 min to fully dissolve the crystals. Subsequently, the optical density (OD) values of each well were measured at 490 nm using an enzyme marker (Tecan infinite M200). Cell adhesion was calculated according to the following formula:

$$\mathrm{Cell\;adhesion\;}\left(\mathrm{\%}\right)=\left[\mathrm{Treat}-\mathrm{Blank}\right]/\left[\mathrm{Control}-\mathrm{Blank}\right]\times 100\%$$

Eeach group had five wells, and the experiment was repeated independent three times.

### M0 macrophage induction and establishment of cell co-culture system

We added PMA (100 ng/ml) to THP-1 for 24 h to induce into M0 macrophages and detected the expression of CD68 mRNA by qRT-PCR to detect the induction effect. After 24 h, M0 macrophages were replaced with fresh RPMI-1640 for 48 h. The HNSCC cells that reached 80% growth density were washed and replaced with serum-free RPMI-1640 medium for 48 h. After 48 h, the medium was collected by centrifugation and the conditioned medium was collected by filtration. Then the medium of 48 h cultured M0 macrophages was discarded and the conditioned medium of HNSCC was added to establish the cell co-culture system.

### Statistical analysis

All statistical analyses were performed using GraphPad Prism 8.0 (GraphPad Software, Inc, CA, USA). All experiments were repeated at least three times independently, and data were expressed as mean ± standard deviation (SD). t-tests were used to analyze differences between two groups, and one-way ANOVA was used to assess differences between at least three groups. Survival curves were plotted using the Kaplan–Meier method, and differences in survival rates were compared using logrank tests. Correlations between two variables were analyzed by Pearson correlation analysis. Univariate and multivariate analysis were performed by Cox regression analysis. Two-tailed *P* values < 0.05 were considered statistically significant.

## Results

### CERCAM is overexpressed in HNSCC

**Fig. 1:**
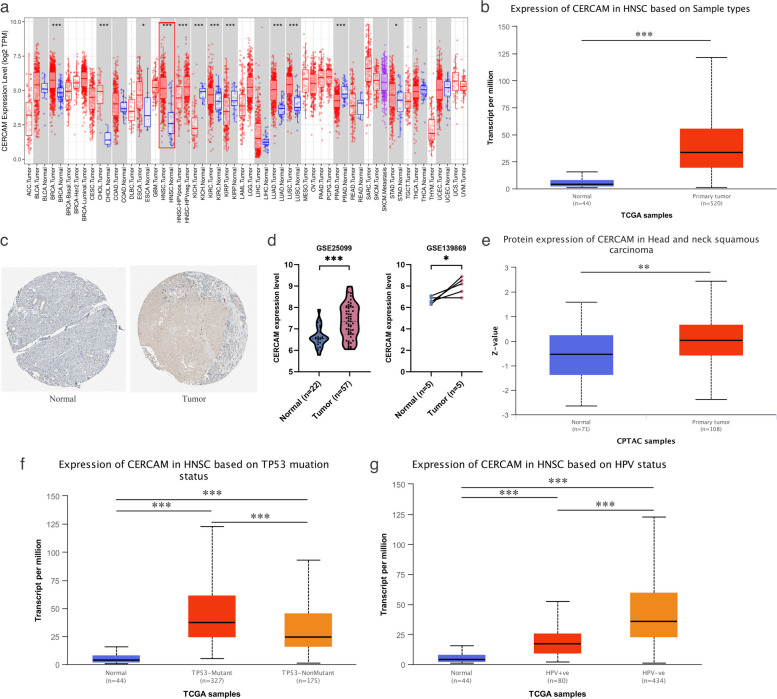
Expression levels of CERCAM in HNSCC. **a** Expression levels of CERCAM at the pan-cancer level, including HNSCC. **b** Differential expression of CERCAM at the mRNA level in HNSCC tumor tissues and their matched normal tissues. **c** Immunohistochemical results of protein level expression differences of CERCAM in HNSCC tumor tissues with their matched normal tissues. **d** Differential expression of CERCAM in HNSCC datasets GSE25099 and GSE139869 in the GEO database in tumor compared to normal tissues. **e** Differences in total protein levels of CERCAM in HNSCC tumor tissues compared to normal tissues. **f** Differential mRNA expression of CERCAM in TP53 mutated compared to TP53 non-mutated HNSCC patients. **g** Differential mRNA expression of CERCAM in HPV( +) compared to HPV(-) HNSCC patients. *P < 0.05, **P < 0.01, ***P < 0.001

As shown in Fig. 1A, we firstly analyzed the differences in the expression levels of CERCAM in various human cancers from the Timer2.0 database and found that CERCAM expression was increased in many tumors, including HNSCC (shown in red box in Fig. 1a). We also examined the difference in mRNA expression of CERCAM in normal and tumor tissues through the TCGA database (Fig. 1b), while the results of IHC using the HPA database further confirmed the overexpression of CERCAM in tumor tissues (Fig. 1c). In order to evaluate the expression of CERCAM better, we used two head and neck squamous carcinoma datasets (GSE25099, GSE139869) from the GEO database and found that CERCAM was also highly expressed in tumor tissues (Fig. 1d). In addition, we used UALCAN database to assess the difference in total protein expression levels of CERCAM in HNSCC and the results were consistent (Fig. 1e). Finally, we further evaluated the difference in CERCAM expression in HNSCC patients with or without TP53 mutation and in HNSCC patients with or without HPV infection, and interestingly CERCAM expression was increased in HNSCC patients with TP53 mutation and HPV negativity (Fig. 1f, g).

### Correlation of CERCAM expression levels with clinicopathological characteristics of HNSCC patients

**Fig. 2:**
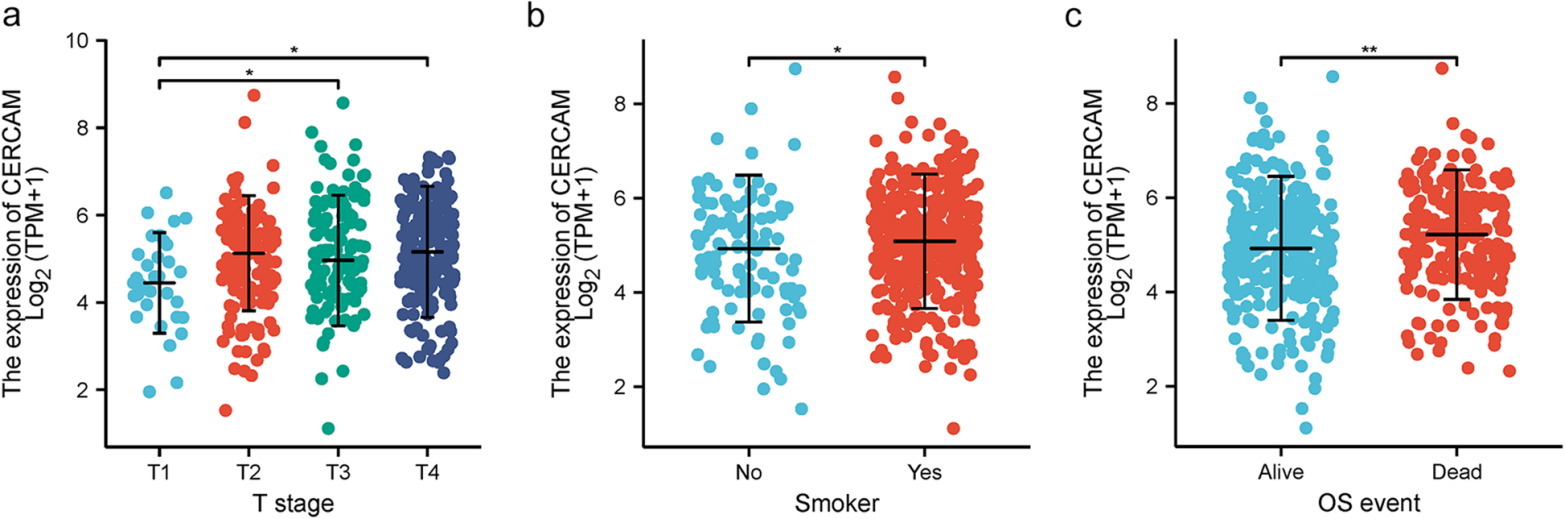
Clinical factors associated with CERCAM expression in HNSCC patients. **a** The expression level of CERCAM correlates with the clinical T-stage of HNSCC patients. **b** The expression level of CERCAM correlates with the history of smoking in HNSCC patients. **c** The expression level of CERCAM was correlated with the survival resolution with OS in HNSCC patients. *P < 0.05, **P < 0.01, ***P < 0.001

We used information on clinicopathological characteristics from the TCGA-HNSCC dataset to examine the correlation between CERCAM expression levels and clinicopathological characteristics of HNSCC patients (as shown in Table 1). Although no differences were observed in the correlation between CERCAM expression and factors such as race, age, gender, clinical grade, pathological grade, and history of alcohol consumption, but significant differences were observed in the correlation with tumor T-stage, smoking history, and survival outcome OS (Fig. 2 a-c).

**Table 1:** Correlation of CERCAM expression levels with clinicopathological characteristics of HNSCC patients

Characteristic	N	Expression of CERCAM	P
OS event			0.008
Alive	284	4.879 ± 1.203	
Dead	218	5.155 ± 1.067	
Gender			ns
Female	134	4.945 ± 1.093	
Male	368	5.018 ± 1.175	
Race			ns
Asian	10	5.185 ± 1.025	
Black or African American	47	5.328 ± 1.227	
White	428	4.938 ± 1.143	
Age			ns
< = 60	245	4.999 ± 1.231	
> 60	256	5.002 ± 1.077	
Smoker			0.044*
No	111	4.798 ± 1.251	
Yes	381	5.047 ± 1.109	
Alcohol history			ns
No	158	4.905 ± 1.157	
Yes	333	5.042 ± 1.154	
Clinical stage			ns
Stage I	19	4.652 ± 1.117	
Stage II	95	5.076 ± 0.929	
Stage III	102	4.872 ± 1.188	
Stage IV	272	5.059 ± 1.212	
T stage			
T1	33	4.463 ± 1.042	
T2	144	4.93 ± 1.12	
T3	131	5.111 ± 1.174	T1vsT3(0.032*)
T4	179	5.106 ± 1.153	T1vsT4(0.010*)
N stage			ns
N0	239	5.018 ± 1.079	
N1	80	4.94 ± 1.216	
N2	154	5.018 ± 1.213	
N3	7	6.089 ± 1.047	
M stage			ns
M0	472	5.004 ± 1.139	
M1	5	4.383 ± 0.576	
Histologic grade			ns
G1	62	4.791 ± 1.016	
G2	300	5.016 ± 1.155	
G3&G4	121	5.091 ± 1.155	
Lymphovascular invasion			ns
No	219	5.025 ± 1.171	
Yes	122	5.139 ± 1.071	

^*^P < 0.05, **P < 0.01, ***P < 0.001

### CERCAM is a potential prognostic and diagnostic marker for HNSCC

**Fig. 3:**
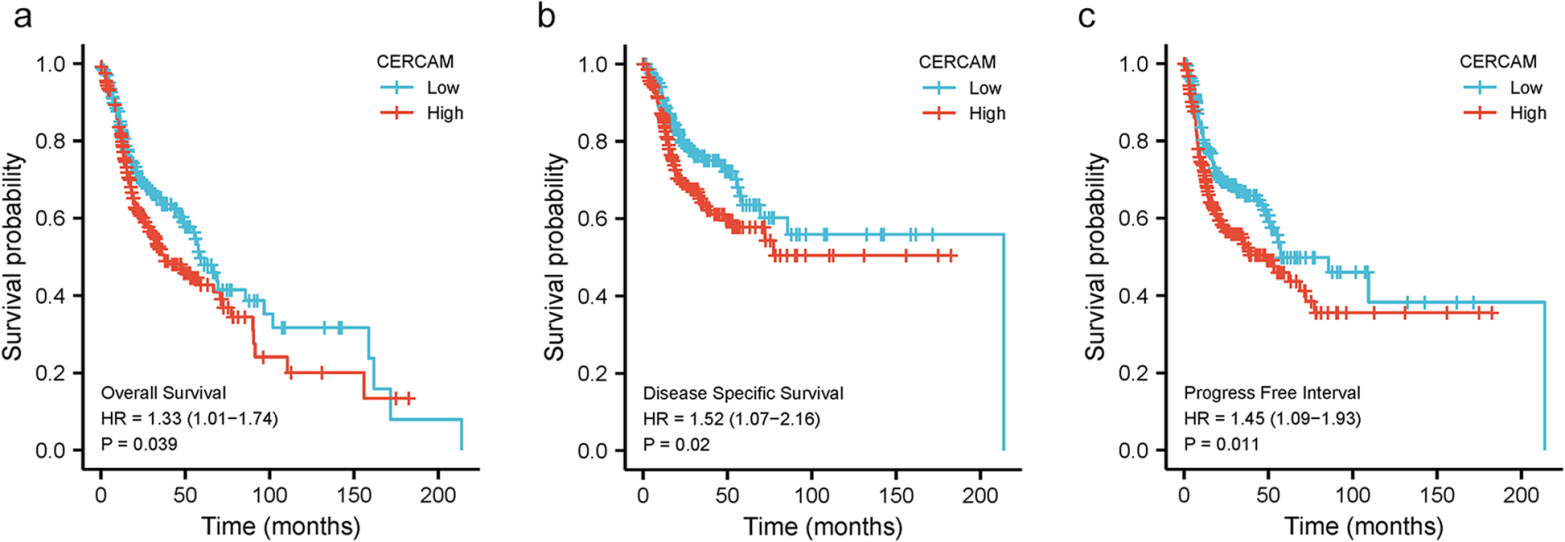
CERCAM is a potential prognostic marker for HNSCC. **a** High expression levels of CERCAM are associated with poor OS in HNSCC patients. **b** High expression levels of CERCAM were associated with poor DSS in HNSCC patients. **c** High expression levels of CERCAM were associated with poor PFI in HNSCC patients. *P < 0.05, **P < 0.01, ***P < 0.001

Based on TCGA-HNSCC data, we analyzed the prognostic value of CERCAM in HNSCC. K-M survival curve analysis showed that HNSCC patients with high CERCAM expression were associated with significantly lower OS (*p* = 0.039), DSS (*p* = 0.02), and PFI (*p* = 0.011) compared to those with low CERCAM expression (as shown in Fig. 3 a-c). As shown in Table 2, the expression level of CERCAM was an independent risk factor for predicting OS, DSS, and PFI by univariate and multivariate COX regression analysis. Meanwhile, clinical N stage was also an independent predictor of OS, DSS, and PFI. In addition, clinical M-stage was an independent predictor of OS and DSS but not PFI. In conclusion, our study showed that CERCAM expression was associated with increased malignancy and poorer prognosis of HNSCC tumors.

**Table 2:** Factors affecting the poor prognosis of HNSCC patients

Characteristics	HR for OS (95% CI)		HR for DSS (95% CI)		HR for PFI (95% CI)	
	**Univariate**	**Multivariate**	**Univariate**	**Multivariate**	**Univariate**	**Multivariate**
Gender (Female vs male)	0.764		0.974		1.058	
Age (< = 60 vs > 60)	1.252		1.078		1.078	
Smoker (No vs Yes)	1.089		1.034		0.892	
Alcohol history (No vs Yes)	0.952		1.212		1.368	
Clinical stage (I&II vs III&IV)	1.217		1.151		1.192	
T stage (T1&T2 vs T3&T4)	1.245		1.459		1.342	
N stage (N0&N1 vs N2&N3)	1.384*	1.352*	1.655**	1.693**	1.446*	1.461*
M stage (M0 vs M1)	4.745**	4.591**	8.056***	10.419***	2.827	
Histologic grade (G1&G2 vs G3&G4)	0.939		1.051		0.970	
CERCAM (Low vs High)	1.330*	1.361*	1.518*	1.609*	1.586**	1.517**

^*^P < 0.05, **P < 0.01, ***P < 0.001

**Fig. 4:**
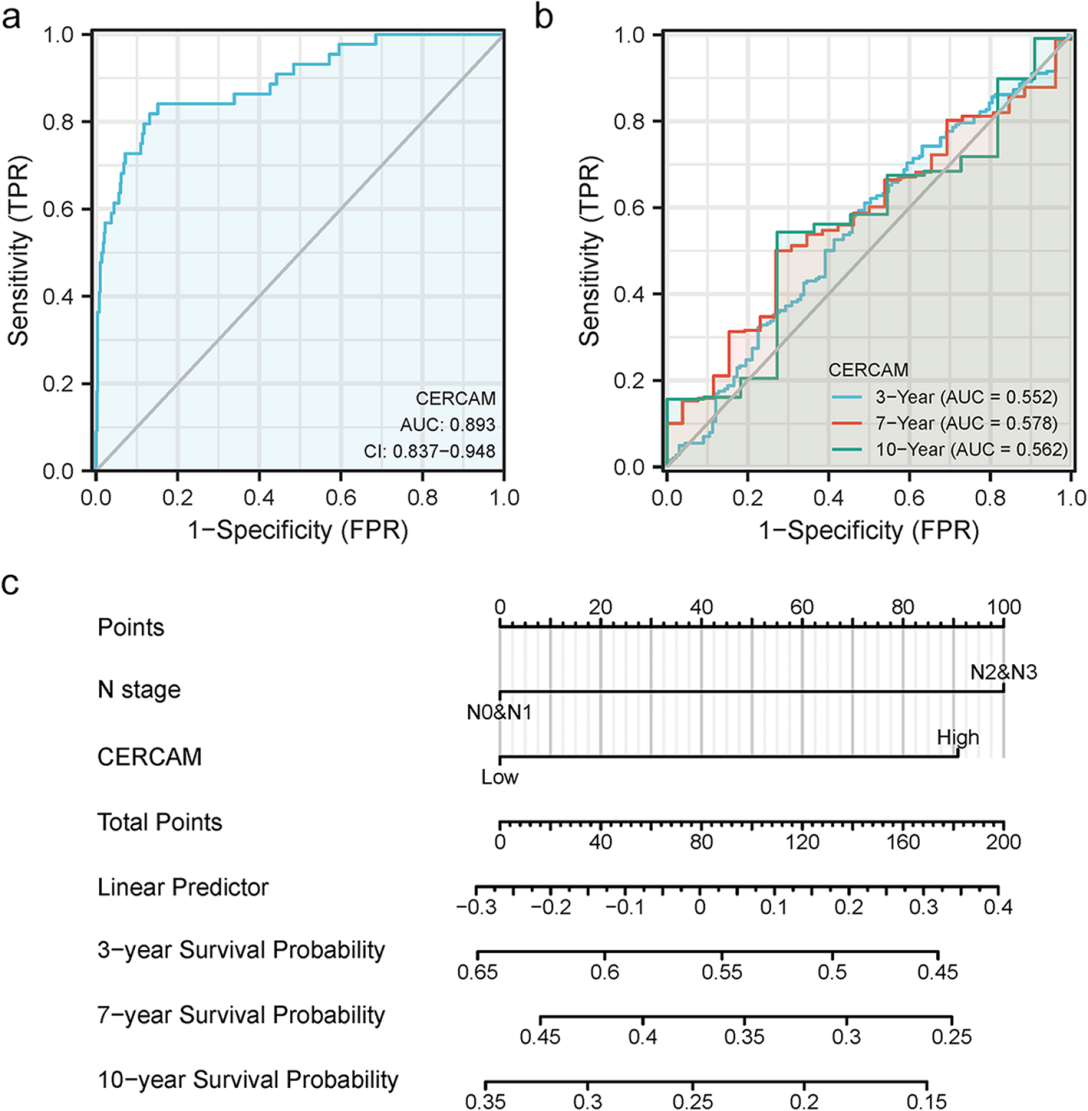
Diagnostic value of CERCAM in HNSCC. **a** Diagnostic ROC curves for differentiating HNSCC tissues and normal tissues based on the expression level of CERCAM. **b** Time-dependent ROC curves for predicting the survival rate of HNSCC patients at 3, 7 and 10 years based on the expression level of CERCAM. **c** Columnar line graph model constructed with clinical N stage and CERCAM expression levels to predict 3, 7, and 10-year survival rates of HNSCC patients

We next assessed the diagnostic value of CERCAM using the ROC curve. As shown in Fig. 4a, the area under the curve (AUC) of CERCAM was 0.893, indicating the satisfactory diagnostic value of CERCAM in HNSCC. The results of time-dependent ROC analysis curves showed that the AUC values for predicting the survival rate of HNSCC patients at 3, 7, and 10 years based on the expression level of CERCAM were above 0.55, as shown in Fig. 4b. In addition, we constructed a column line graph model including tumor clinical N stage and CERCAM expression level as factors (Fig. 4c). Based on univariate and multivariate Cox regression analysis, clinical N stage and CERCAM expression level were valuable independent prognostic predictors for OS, DSS and PFI. The column line graph model showed that the above factors showed high clinical significance in predicting the survival probability of HNSCC patients at 3, 7, and 10 years.

### Prognostic significance of CERCAM in a clinicopathological subgroup of HNSCC patients

**Fig. 5:**
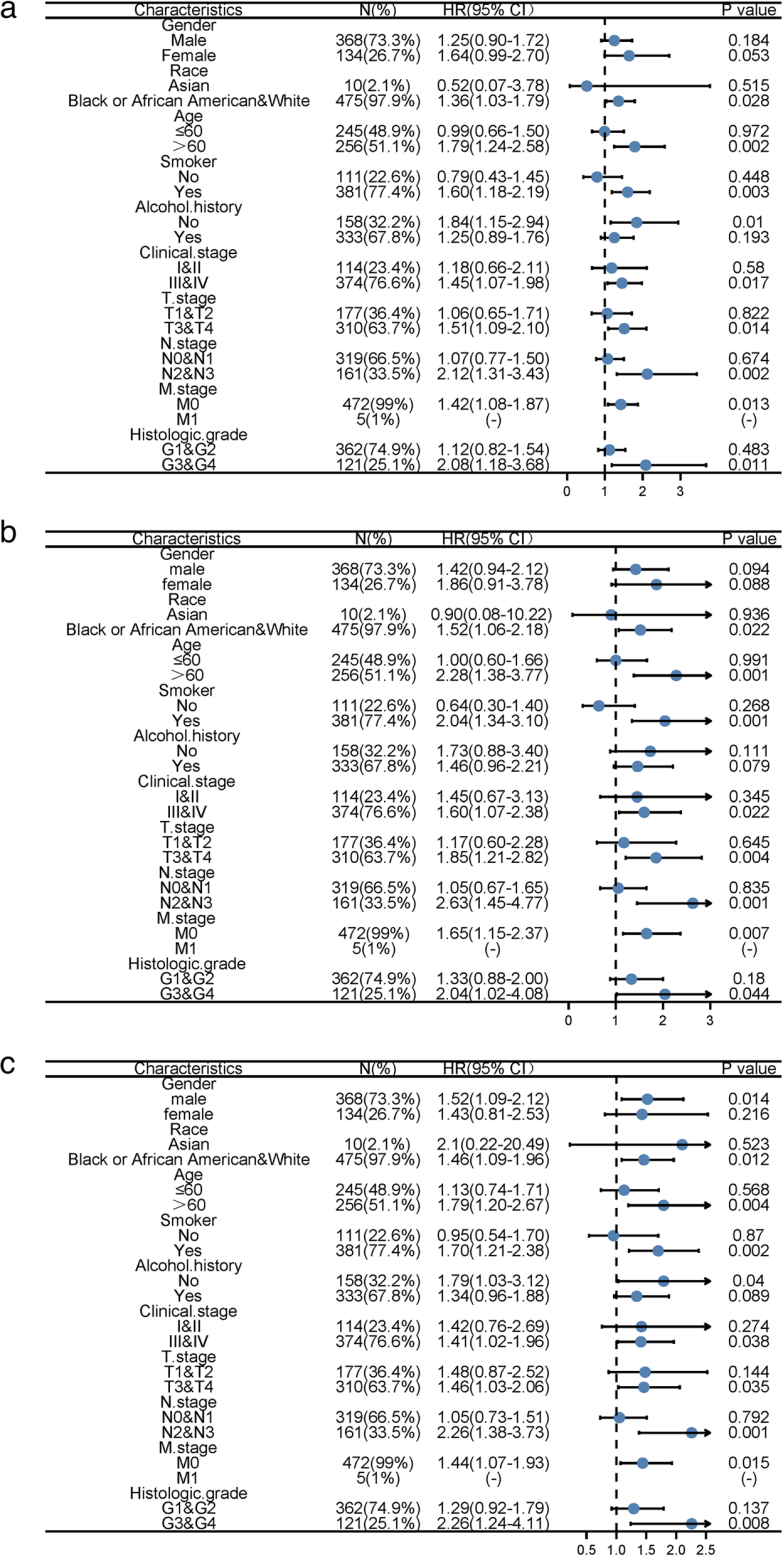
Prognostic forest plot of CERCAM in clinicopathological subgroups of patients with HNSCC. **a** Prognostic levels of OS for CERCAM in the HNSCC clinicopathology subgroup. **b** Prognostic levels of DSS for CERCAM in the HNSCC clinicopathology subgroup of patients. **c** Prognostic level of PFI for CERCAM in the clinicopathological subgroup of patients with HNSCC. *P < 0.05, **P < 0.01, ***P < 0.001

To investigate the prognostic impact of CERCAM expression in clinicopathological subgroups of HNSCC patients, we did Cox regression analysis of OS, DSS, and PFI values for each subgroup of patients in TCGA-HNSCC, and the results are shown in the form of forest plots (Fig. 5), and information on specific statistical values of OS, DSS, and PFI for each clinicopathological subgroup of patients is shown in Table 3. High CERCAM expression levels were associated with poor PFI in male patients. In race, its high expression could be found to be associated with poor OS, DSS, and PFI in both blacks and whites. Age was a significant factor affecting prognosis, and the results showed that high expression of CERCAM in patients over the age of 60 years was associated with poor OS, DSS, and PFI. In patients with a history of smoking, high expression of CERCAM was associated with poor OS, DSS, and PFI. In patients without a history of alcohol consumption high CERCAM expression was only associated with poor OS, PFI. In contrast, high expression levels of CERCAM in patients with advanced T3 and T4, patients with lymph node involvement in stages N2 and N3, and M0 patients without distant metastases were found to be associated with poor OS, DSS, and PFI in clinical T, N, and M stages. Finally, high expression levels of CERCAM in histologically graded G3 and G4 patients were correlated with poor OS, DSS, and PFI.

**Table 3:** Prognostic significance of CERCAM in the clinicopathological subgroup of patients with HNSCC

Characteristics	N (%)	HR for OS (95% CI)	HR for DSS (95% CI)	HR for PFI (95% CI)
Gender				
Male	368(73.3%)	1.25(0.90–1.72)	1.42(0.94–2.12)	1.52(1.09–2.12)*
Female	134(26.7%)	1.64(0.99–2.70)	1.86(0.91–3.78)	1.43(0.81–2.53)
Race				
Asian	10(2.1%)	0.52(0.07–3.78)	0.90(0.08–10.22)	2.1(0.22–20.49)
Black or African American&White	475(97.9%)	1.36(1.03–1.79)*	1.52(1.06–2.18)*	1.46(1.09–1.96)*
Age				
≤ 60	245(48.9%)	0.99(0.66–1.50)	1.00(0.60–1.66)	1.13(0.74–1.71)
> 60	256(51.1%)	1.79(1.24–2.58)**	2.28(1.38–3.77)**	1.79(1.20–2.67)**
Smoker				
No	111(22.6%)	0.79(0.43–1.45)	0.64(0.30–1.40)	0.95(0.54–1.70)
Yes	381(77.4%)	1.60(1.18–2.19)**	2.04(1.34–3.10)**	1.70(1.21–2.38)**
Alcohol.history				
No	158(32.2%)	1.84(1.15–2.94)*	1.73(0.88–3.40)	1.79(1.03–3.12)*
Yes	333(67.8%)	1.25(0.89–1.76)	1.46(0.96–2.21)	1.34(0.96–1.88)
Clinical.stage				
I&II	114(23.4%)	1.18(0.66–2.11)	1.45(0.67–3.13)	1.42(0.76–2.69)
III&IV	374(76.6%)	1.45(1.07–1.98)*	1.60(1.07–2.38)*	1.41(1.02–1.96)*
T.stage				
T1&T2	177(36.4%)	1.06(0.65–1.71)	1.17(0.60–2.28)	1.48(0.87–2.52)
T3&T4	310(63.7%)	1.51(1.09–2.10)*	1.85(1.21–2.82)**	1.46(1.03–2.06)*
N.stage				
N0&N1	319(66.5%)	1.07(0.77–1.50)	1.05(0.67–1.65)	1.05(0.73–1.51)
N2&N3	161(33.5%)	2.12(1.31–3.43)**	2.63(1.45–4.77)**	2.26(1.38–3.73)**
M.stage				
M0	472(99%)	1.42(1.08–1.87)*	1.65(1.15–2.37)**	1.44(1.07–1.93)*
M1	5(1%)	(-)	(-)	
Histologic.grade				
G1&G2	362(74.9%)	1.12(0.82–1.54)	1.33(0.88–2.00)	1.29(0.92–1.79)
G3&G4	121(25.1%)	2.08(1.18–3.68)**	2.04(1.02–4.08)*	2.26(1.24–4.11)**

^*^P < 0.05, **P < 0.01, ***P < 0.001

### Silencing CERCAM contributes to inhibit the malignant progression of HNSCC cells

**Fig. 6:**
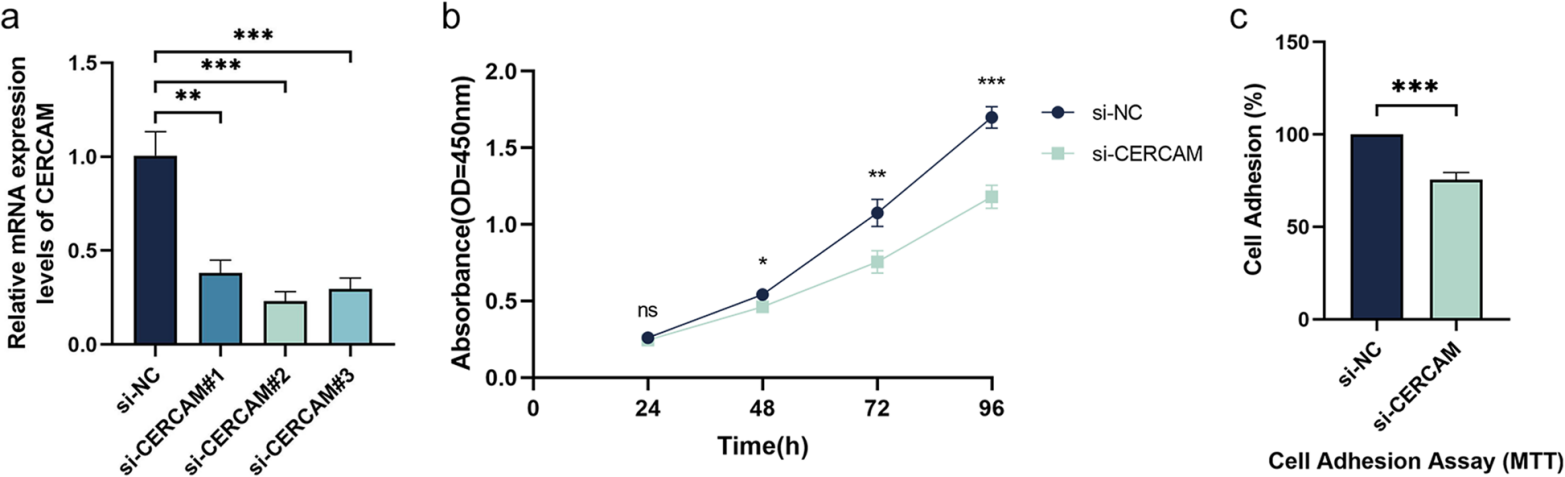
Silencing CERCAM to inhibit malignant progression of HNSCC cells. **a** Transfection efficiency assay of CERCAM. **b** Differences in proliferation levels of HNSCC cells by CCK-8 assay. **c** Differences in the adhesion ability of HNSCC cells were detected by cell adhesion assay. The results are presented as the mean ± SD, *P < 0.05, **P < 0.01, ***P < 0.001

These above results suggest that CERCAM has biological properties that promote the malignant progression of HNSCC. To investigate whether suppressing the expression of this gene contributes to inhibit the malignant progression of HNSCC, and provide a theoretical basis for distant gene therapy. As shown in Fig. 6a, by cell transfection assay, we used small interfering RNA to knock down the expression of CERCAM in HNSCC cells, and the results of qRT-PCR showed that si-CERCAM#2 transfection was the most efficient and the mRNA expression level of CERCAM was the lowest. Therefore, we selected si-CERCAM#2 for subsequent experiments. We then examined the effect of CERCAM expression in HNSCC cells on cell proliferation by CCK-8 assay, and the results are shown in Fig. 6b. Compared with the control group, the proliferation level of HNSCC cells decreased with the silencing of CERCAM, and the difference was significant (24 h:ns,48 h:*P* < 0.05, 72 h:*P* < 0.01, 96 h:*P* < 0.001). Subsequently, we detected the effect of CERCAM expression on cell adhesion ability in HNSCC cells by cell adhesion assay using MTT method, and the results are shown in Fig. 6c. Compared with the control group, the adhesion ability of HNSCC cells was decreased after silencing CERCAM, and the difference was significant (*P* < 0.001). Therefore, silencing CERCAM helps to inhibit the malignant progression of HNSCC cells.

#### CERCAM mutations in HNSCC tumors

**Fig. 7:**
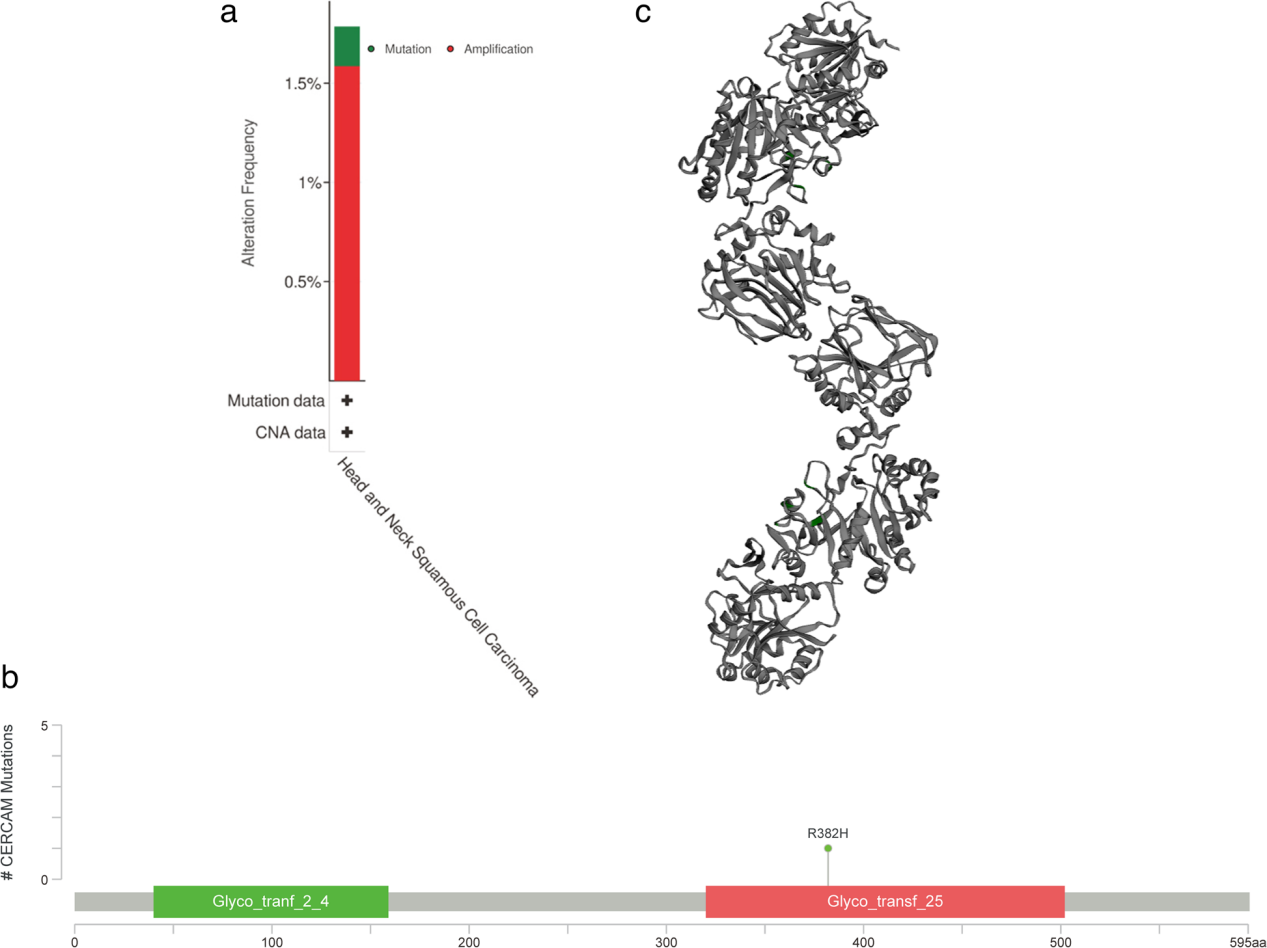
Mutations of CERCAM gene in HNSCC. **a** Frequency of gene changes in CERCAM in HNSCC. **b** CERCAM mutation site in HNSCC. **c** The R382H mutation site is shown in the 3D protein structure of CERCAM

To explore CERCAM mutations in HNSCC, we used the cBioPortal platform to analyze its genetic change status based on TCGA data. The results showed the high CERCAM amplification in HNSCC (Fig. 7a). Gene mutation analysis identified missense as its gene mutation type, we found missense mutation R382H within the Glyco_transf_25 domain was mutated (Fig. 7b). Figure 7c also shows the 3D structure of the protein with the R382H mutation in CERCAM.

#### Analysis of CERCAM methylation levels in HNSCC tumors

**Fig. 8:**
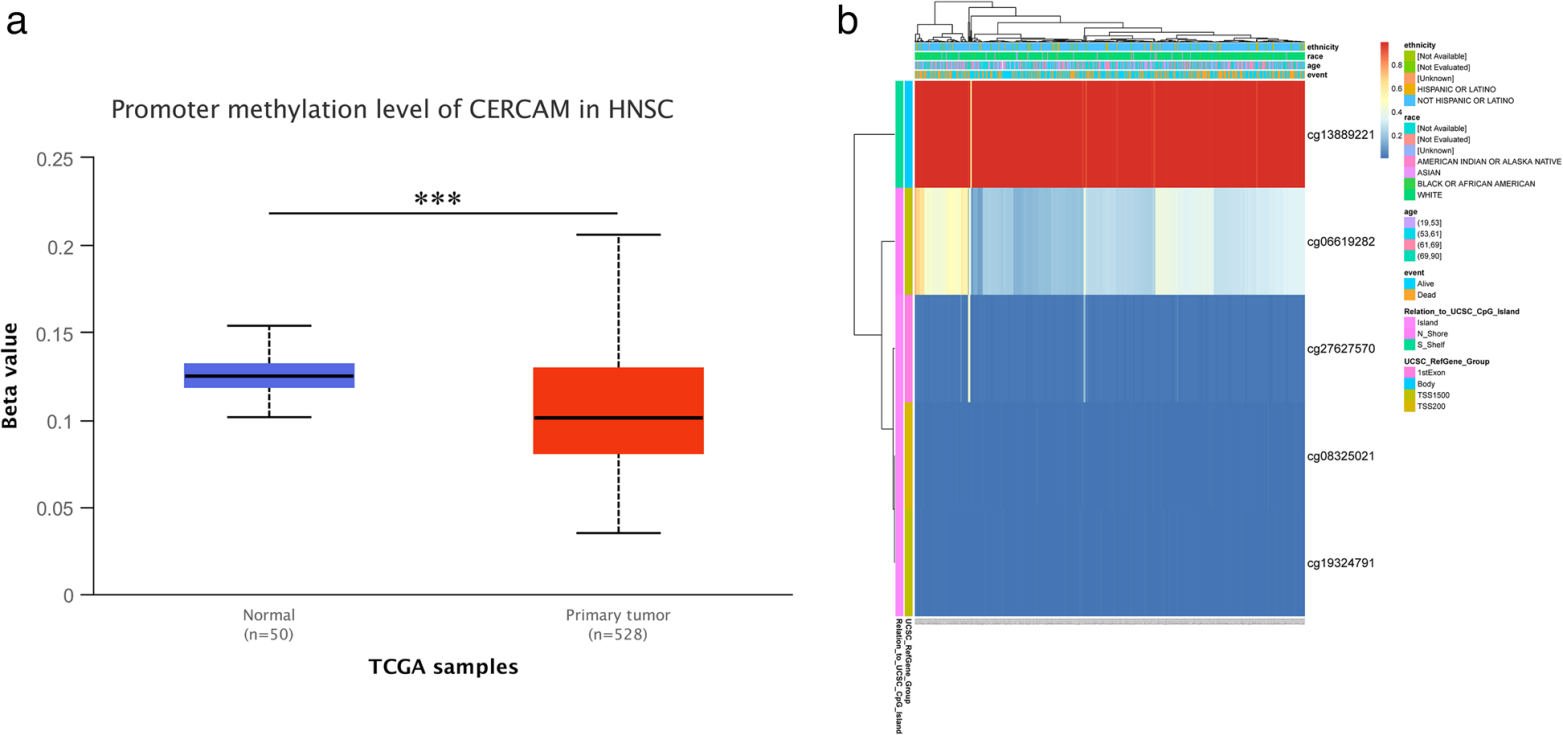
Analysis of CERCAM methylation levels in HNSCC tumors. **a** CERCAM methylation levels are decreased in tumor tissues. **b** Heat map of DNA methylation levels in CERCAM gene analyzed by MethSurv platform. *P < 0.05, **P < 0.01, ***P < 0.001

We first used the UALCAN platform to analyze the DNA methylation levels of CERCAM genes in patient tumor tissues with their matched normal tissues in the TCGA-HNSCC dataset (Fig. 8a), we found that the CERCAM gene showed lower methylation levels in tumor tissues, with a significant difference (*P* < 0.05). We then applied the MethSurv platform to visually analyze the DNA methylation levels of CPG islands in the CERCAM gene, and we found that five CPG islands including cg13889221, cg06619282, cg27627570, cg08325021, and cg19324791 showed altered methylation levels (Fig. 8b). Combined with the above results that CERCAM expression correlated with poorer prognosis, suggesting that hypomethylation levels of CERCAM in tumors are accompanied with poorer prognosis, we hypothesize that CERCAM may function as an oncogene in tumors.

### Correlation analysis of CERCAM and immune cell infiltration in HNSCC

**Fig. 9:**
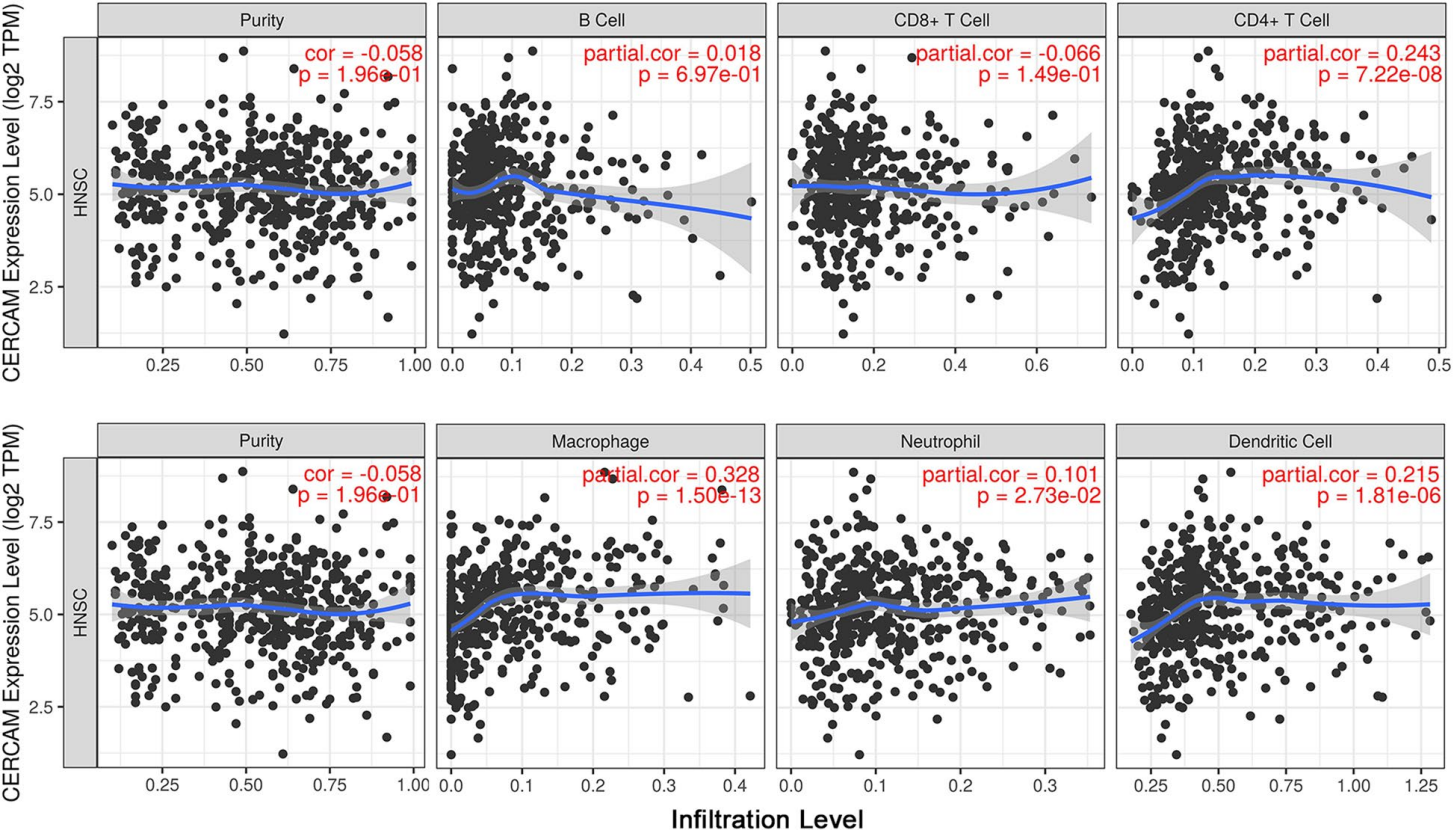
CERCAM correlates with immune cell infiltration in HNSCC. CERCAM expression was most significantly correlated with macrophages in tumors

Many studies have shown that immune cells in the tumor microenvironment are involved in regulating the process of tumor development. Based on this, we used the Timer database to explore the correlation between CERCAM expression and immune cell infiltration in HNSCC.As shown in Fig. 9, we found that the expression of CERCAM correlated most significantly with the infiltration of macrophages in tumors (Cor = 0.328, *P* = 1.5e-13), followed by CD4^+^ T cells (Cor = 0.243, *P* = 7.22e-8) and DC cells (Cor = 0.215, *P* = 1.81e-6). However, the expression level of CERCAM correlated weakly or not significantly with B cells, CD8^+^ T cells, and neutrophils in tumors.

To further investigate the correlation between CERCAM and different types of immune cell subpopulations, we analyzed the relationship between CERCAM expression in HNSCC and immune markers of different types of immune infiltrating cells (as shown in Table 4).The expression levels of CERCAM significantly correlated with marker genes of B cells, NK cells, monocytes, centrophages, DC cells, macrophages, M2 type macrophages, and T cells (including CD8 + T cells, Th1, Th2, Th9, Th17, Th22, and Tregs). And no significant correlation or weak correlation with Tfh and M1 macrophage marker genes.

**Table 4:** Correlation between CERCAM and different types of immune cell subpopulation marker genes

Cell Type	Gene marker	None		Purity		Cell type	Gene marker	None		Purity	
		**Cor**	**P**	**Cor**	**P**			**Cor**	**P**	**Cor**	**P**
B cell	CD19	-0.156	***	-0.165	***		IL23R	-0.053		-0.045	
	CD20(KRT20)	-0.033		0.003			IL17A	-0.158	***	-0.155	***
	CD38	-0.028		-0.008		Th22	CCR10	0.063		0.075	
NK cell	XCL1	0.114	**	0.128	**		AHR	0.3	***	0.296	***
	CD7	-0.046		-0.054		Treg	FOXP3	0.144	***	0.149	***
	KIR3DL1	-0.116	**	-0.114	*		CD25(IL2RA)	0.22	***	0.227	***
CD8 + T cell	CD8A	-0.116	**	-0.119	**		CCR8	0.173	***	0.181	***
	CD8B	-0.108	*	-0.108	*	Macrophage	CD68	0.291	***	0.273	***
Tfh	BCL6	0.003		0.012			CD11b(ITGAM)	0.141	**	0.144	**
	ICOS	0.055		0.061		M1	INOS(NOS2)	-0.086	*	-0.068	
	CXCR5	-0.08		-0.089	*		IRF5	-0.009		-0.013	
TH1	T-bet(TBX21)	-0.077		-0.078			COX2(PTGS2)	-0.074		-0.058	
	STAT4	0.228	***	0.239	***	M2	CD163	0.309	***	0.293	***
	IL12RB2	0.037		0.024			VSIG4	0.351	***	0.336	***
	WSX1(IL27RA)	0.162	***	0.184	***		CD206(MRC1)	0.388	***	0.322	***
	STAT1	0.044		0.029		TAM	CCL2	0.255	***	0.26	***
	IFN-γ(IFNG)	-0.17	***	-0.173	***		CD80	0.238	***	0.248	***
	TNF-α(TNF)	0.072		0.085			CD86	0.255	***	0.264	***
Th2	GATA3	0.138	**	0.14	**		CCR5	0.01		0.014	
	CCR3	0.233	***	0.235	***	Monocyte	CD14	0.265	***	0.257	***
	STAT6	-0.005		-0.004			CD16(Fcgr3B)	0.11	*	0.092	*
	STAT5A	0.001		0.011			CD115(CSF1R)	0.297	***	0.303	***
Th9	TGFBR2	0.473	***	0.464	***	Neutrophil	CD66b	-0.112	*	-0.097	*
	IRF4	-0.042		-0.045			CD15(FUT4)	0.341	***	0.356	***
	PU.1(SPI1)	0.231	***	0.235	***	DC cell	CD1C	0.086	*	0.092	*
Th17	STAT3	-0.052		-0.045			CD141(THBD)	0.054		0.04	
	IL21R	0.076		0.078			CD11C(ITGAX)	0.211	***	0.207	***

^*^P < 0.05, **P < 0.01, ***P < 0.001

### CERCAM induces macrophage M2 polarization immune infiltration in HNSCC

**Fig. 10:**
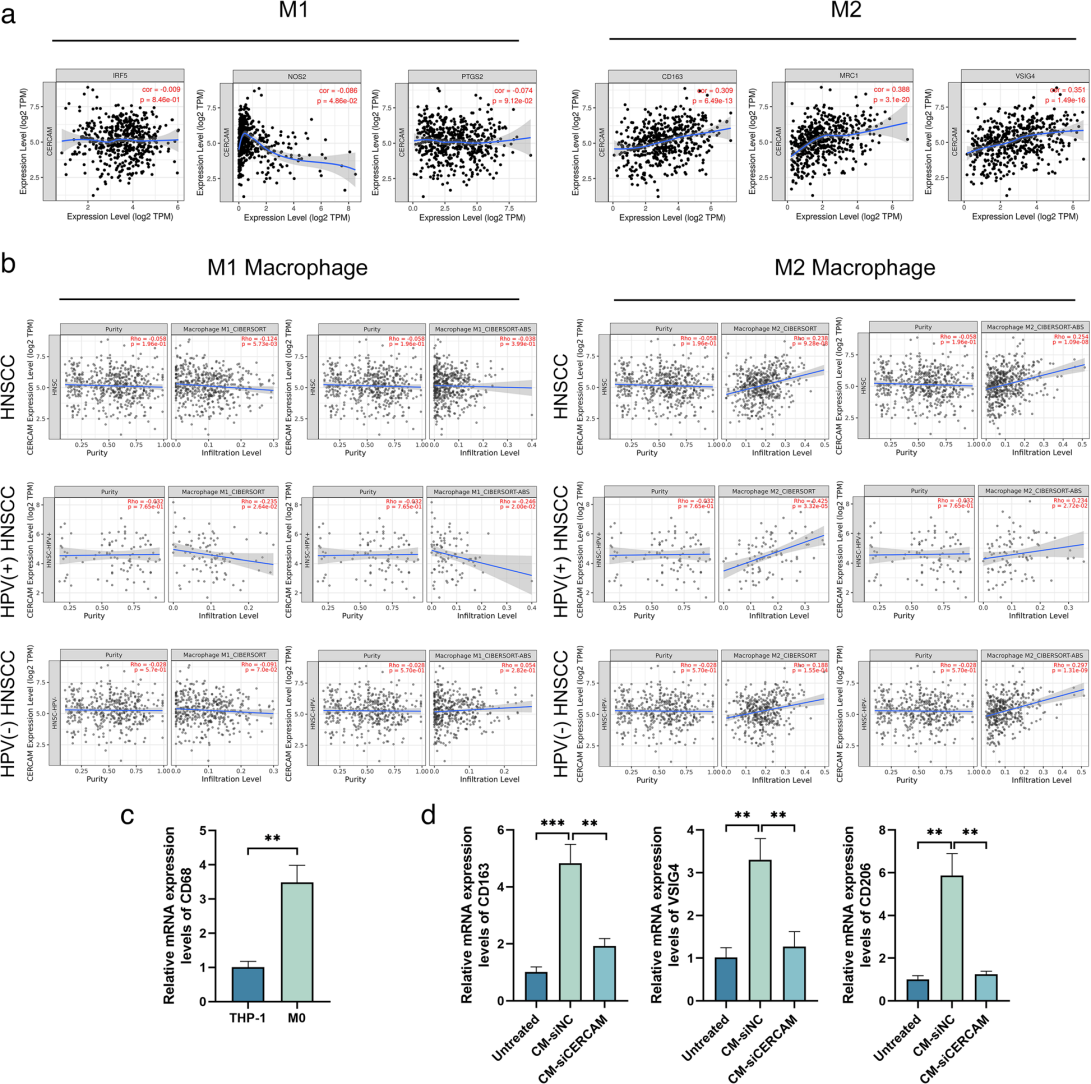
CERCAM induces macrophage M2 polarization immune infiltration in HNSCC. **a** Correlation analysis of CERCAM expression levels in HNSCC with M1 macrophage and M2 macrophage marker genes. **b** Correlation analysis of CERCAM expression levels in HNSCC with M1 and M2 macrophages based on CIBERSOFT and CIBERSOFT-ABS algorithms. **c** Induction of THP-1 into M0 macrophages. **d** Expression levels of genes associated with M2 macrophages were examined to represent the polarization levels. The results are presented as the mean ± SD, *P < 0.05, **P < 0.01, ***P < 0.001

The above results revealed that the expression level of CERCAM correlated most significantly with macrophages in immune infiltrating cells, and the correlation analysis of cellular marker gene expression in its subpopulation revealed that CERCAM correlated only with M2 macrophages, but not with M1 macrophages (Fig. 10a), this is an interesting finding. So, as shown in Fig. 10b, we further explored the correlation of CERCAM expression with the infiltration levels of M1macrophages and M2 macrophages in HNSCC, HPV( +)-HNSCC, and HPV(-)-HNSCC patients with using two algorithms based on CIBERSOFT and CIBERSOFT-ABS in the TIMER2.0 database. We found that in all three types of HNSCC patients, the results of both algorithms showed a significant positive correlation between the expression level of CERCAM and the infiltration level of M2 macrophages, while there was no significant correlation with M1 macrophages. Therefore, we conjecture that CERCAM may be involved in inducing macrophage M2 polarization immune infiltration in HNSCC.

To verify our conjecture, we induced THP-1 into M0 macrophages in vitro (Fig. 10c), M0 macrophages in a separate group as a negative control, and then established a co-culture system of HNSCC cells and M0 macrophages, knockdown of CERCAM gene expression levels in HNSCC by transfection experiments and compared to the si-NC group, then tested the expression levels of M2 macrophage marker genes (CD163, CD206, VSIG4), which represent the levels of induced polarization. As shown in Fig. 10d, we found that the si-CERCAM group had significantly lower mRNA expression levels of CD163, CD206, and VSIG4 genes compared to the NC group, and the difference was significant(P < 0.05). Therefore, the above experimental results suggest that CERCAM in HNSCC may induce macrophages to polarize toward M2.

### Single-cell level expression pattern of CERCAM in HNSCC

**Fig. 11:**
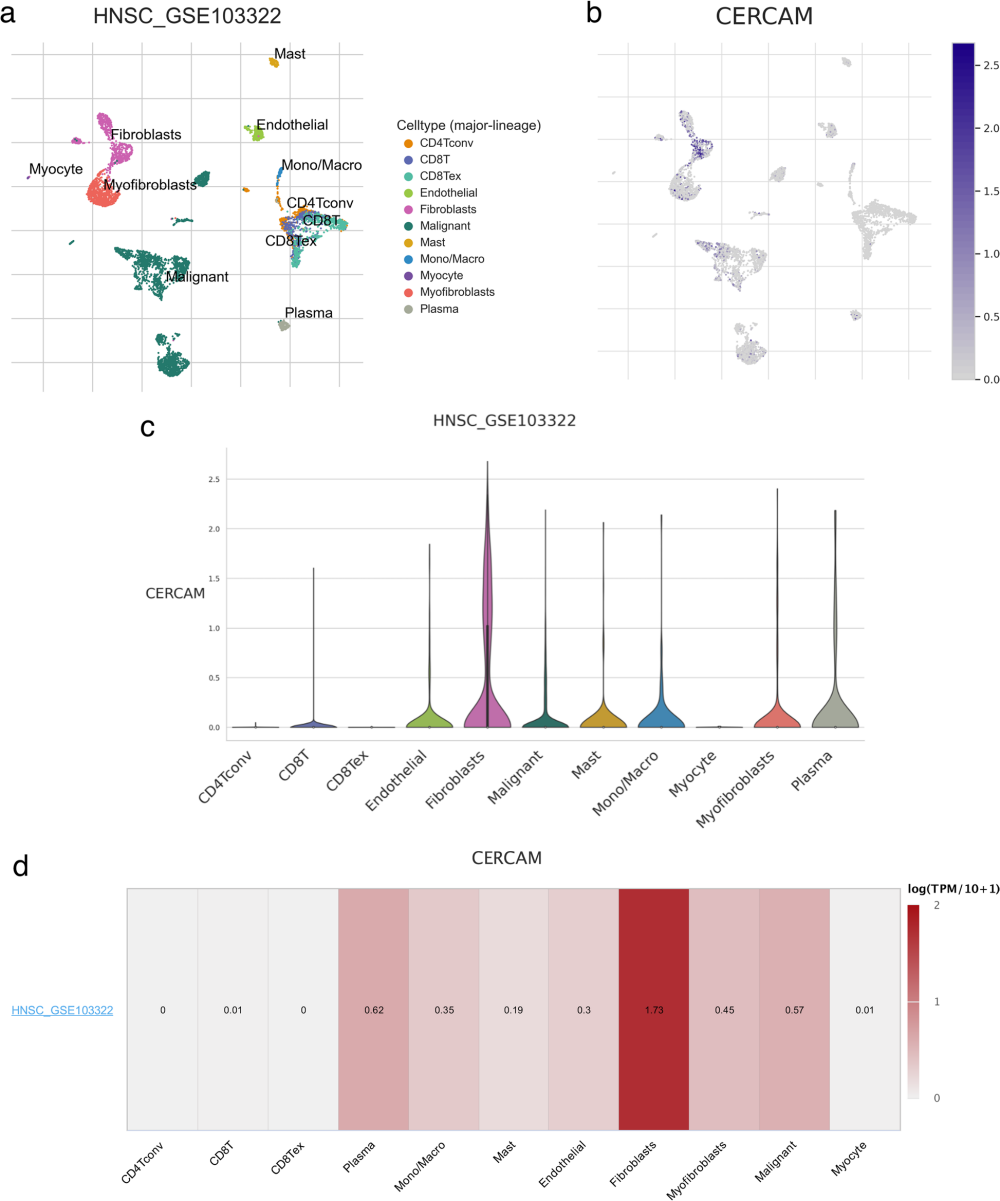
Single-cell level expression pattern of CERCAM in HNSCC. **a** TSNE map of HNSCC single cell sequencing for downscaling visualization. **b** TSNE of CERCAM expression at the single-cell level in HNSCC. **c** Violin plot of CERCAM expression at the HNSCC single cell level. **d** Heat map of CERCAM expression at the HNSCC single-cell level

Single-cell transcriptome sequencing is an emerging technology that has been developed in recent years and can clearly demonstrate gene expression patterns at the single-cell level, enabling us to better understand the differences in gene expression patterns between different single cells in the tumor microenvironment. Therefore, we used the TISCH2 platform to analyze the expression pattern of CERCAM at the single cell level based on one of the largest HNSCC single cell transcriptome sequencing datasets (HNSC_GSE103322) in the GEO database. As shown in Fig. 11.a-d, it was found that CERCAM was most expressed in fibroblasts, plasma cells, and tumor cells, followed by expression in myofibroblasts, monocytes/macrophages, endothelial cells, and mast cells, while no or no significant expression in CD4convT cells, CD8T cells, CD8Tex, and Myocyte.

### CERCAM gene co-expression analysis in HNSCC and PPI network construction with Hub gene screening

**Fig. 12:**
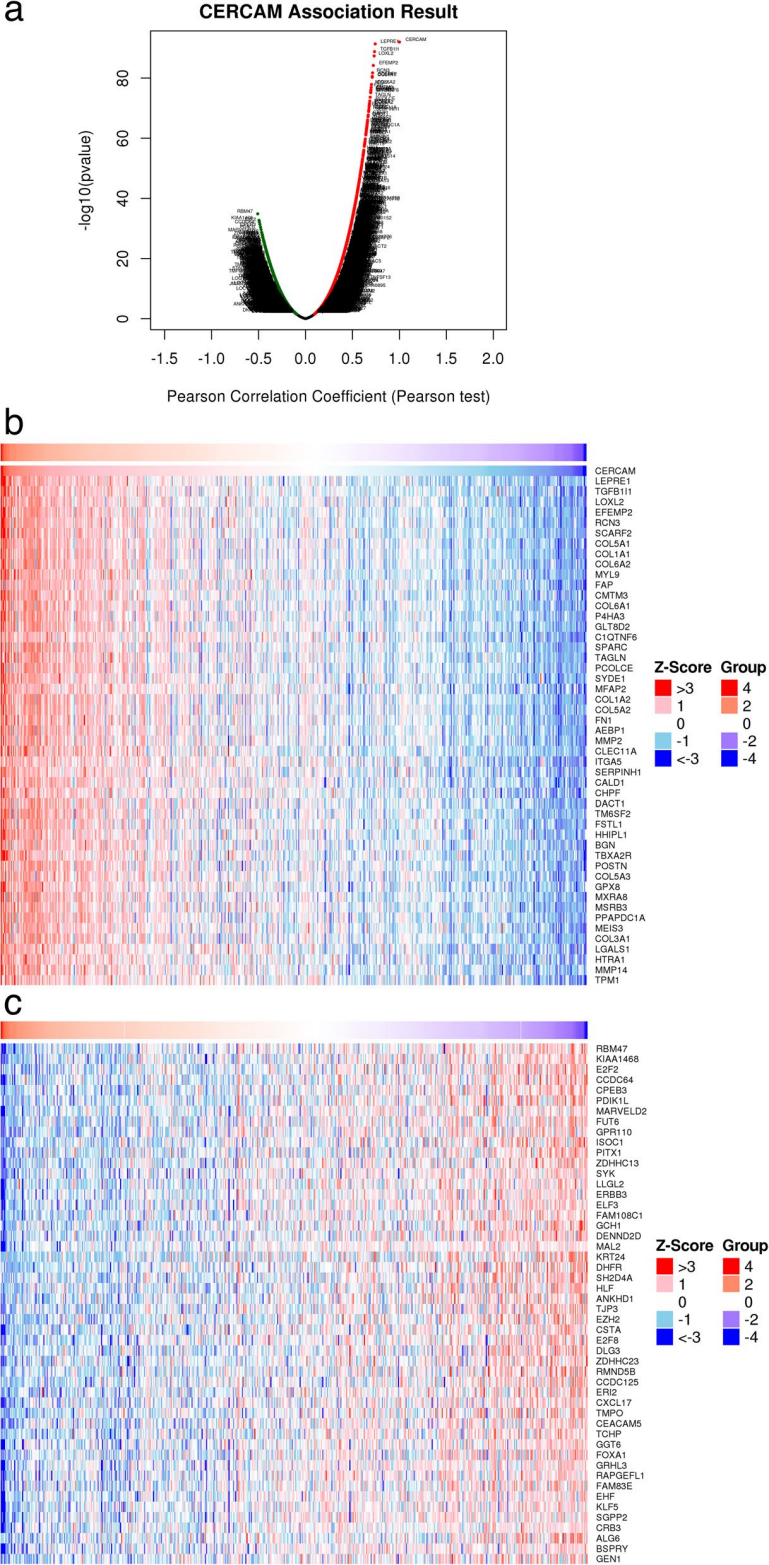
CORECAM gene co-expression analysis in HNSCC. **a** Heat map of volcanoes significantly associated with CERCAM in HNSCC. **b** Heat map of the top 50 genes positively associated with CERCAM co-expression in HNSCC. **c** Heat map of the top 50 genes negatively correlated with CERCAM co-expression in HNSCC

We next analyzed the co-expressed genes with CERCAM in HNSCC to further explore the mechanisms associated with its involvement in cancer. We used the LinkedOmics database based on TCGA-HNSCC data to test genes associated with CERCAM expression by Pearson, and we obtained a total of 11,087 genes associated with CERCAM expression (false discovery rate [FDR] < 0.05). Positive correlations (red dots) and negative correlations (green dots) are shown in Fig. 12a. The top50 genes significantly positively and negatively correlated with CERCAM are shown in Fig. 12b and c.

**Fig. 13:**
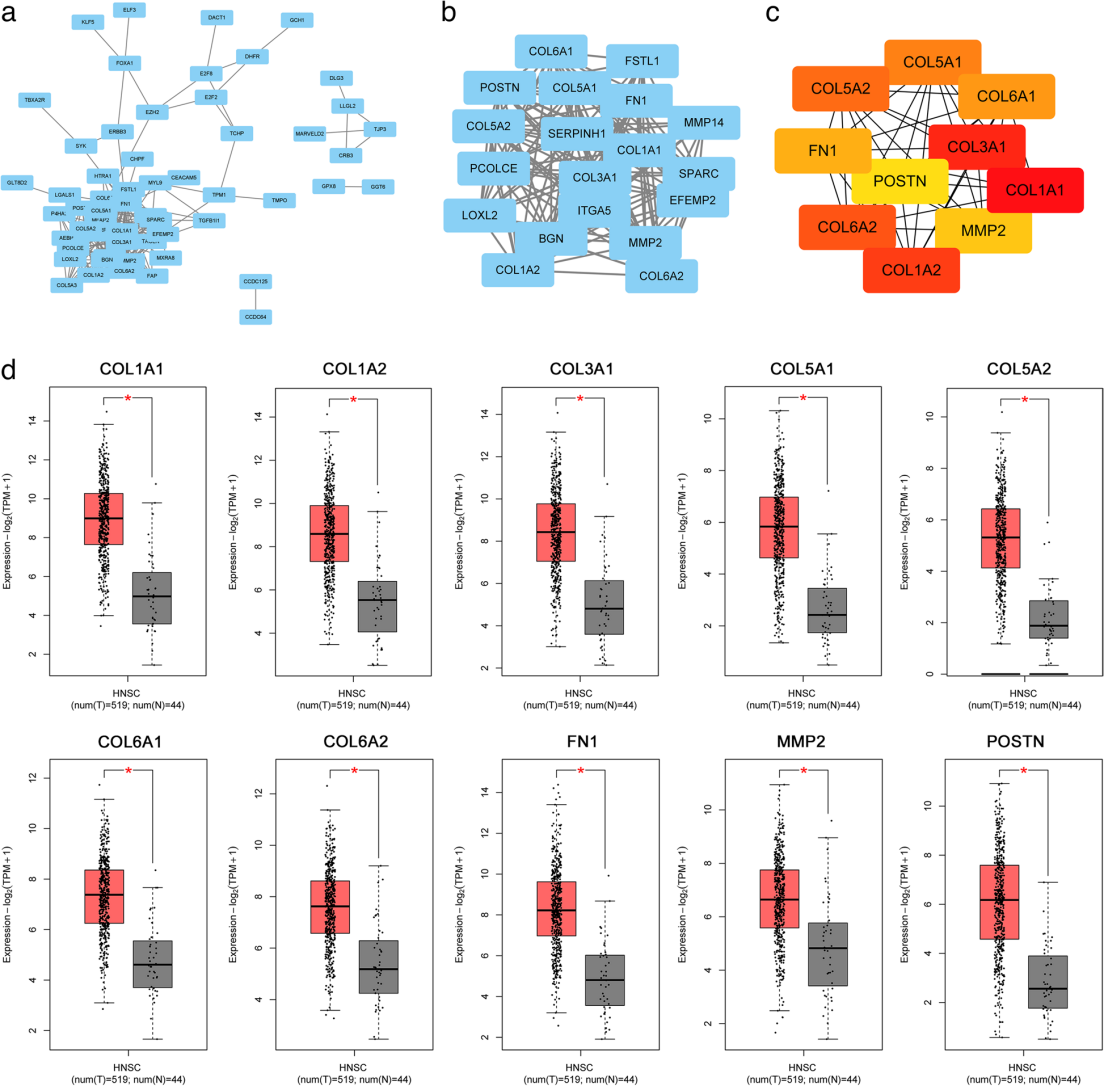
PPI network and module analysis. **a** PPI networks of significantly co-expressed genes. **b** Application of MCODE plug-in to extract the most important modules in the co-expressed gene network. **c** hub gene screening. **d** Differential expression of hub genes in HNSCC tissues versus normal tissues. *P < 0.05, **P < 0.01, ***P < 0.001

We then used Cytoscape software to construct PPI networks from these above strongest co-expressed genes, and the PPI networks consisted of 58 nodes and 265 edges (Fig. 13a). We then applied Cytoscape's MCODE plugin to extract the most important modules among them, including 19 nodes and 153 edges, as shown in Fig. 13b (Score = 12.42). We then applied the cytoHubba plugin to screen 10 hub genes, including COL1A1, COL1A2, COL3A1, COL5A1, COL5A2, COL6A1, COL6A2, FN1, MMP, and POSTN (Fig. 13c). As shown in Fig. 13.d, we further examined the expression of these hub genes in the TCGA-HNSCC dataset, and we found that these genes were all highly expressed in tumor tissues compared to normal tissues.

### CERCAM gene enrichment analysis in HNSCC

**Fig. 14:**
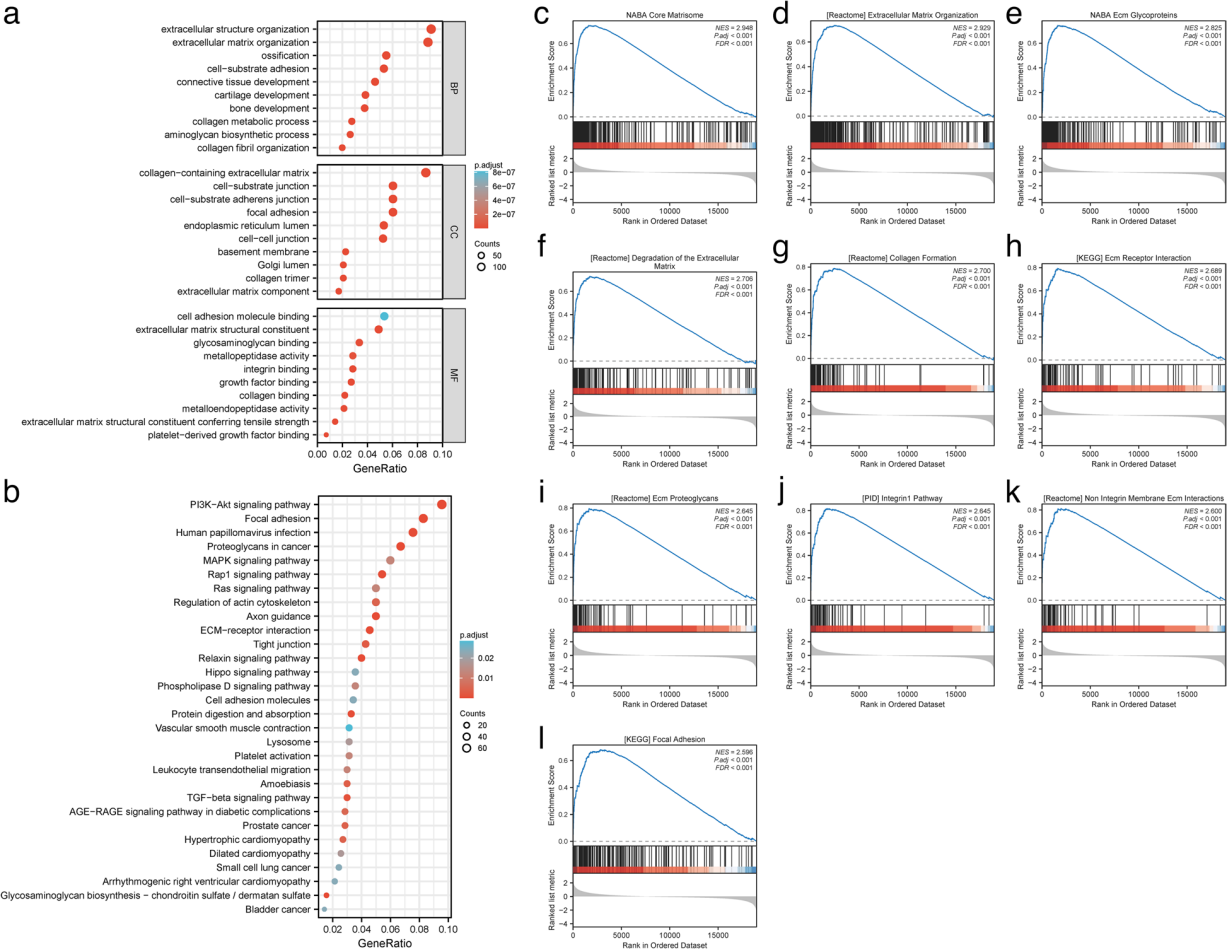
CERCAM gene enrichment analysis in HNSCC. **a** GO enrichment analysis of CERCAM in HNSCC. **b** KEGG enrichment analysis of CERCAM in HNSCC. **c**-**l** GSEA enrichment analysis of CERCAM in HNSCC. *P < 0.05, **P < 0.01, ***P < 0.001

We selected the genes with Pearson coefficient greater than 0.3 among the above 11,087 genes associated with CERCAM expression and obtained a total of 1931 co-expressed genes. We subjected these genes to KEGG and GO enrichment analysis to identify the functions and pathways of CERCAM co-expressed genes involved in HNSCC. As shown in Fig. 14a, the results of functional enrichment analysis revealed significant correlations with functions related to cell adhesion function, including "extracelluar structure organization", "extracelluar matrix organization", "collagen-containing extracellular matrix", "cell-substrate junction ", "cell-substrate adherens junction", "focal adhesion", "cell adhesion molecule binding", "extracelluar matrix structural constituent". As shown in Fig. 14b, the results of the pathway enrichment analysis showed that co-expressed genes were mainly enriched in cell adhesion-related pathways including "Focal adhesion" and "Rap1", and we also found that they were most enriched in "PI3K-Akt" and "MAPK". Nowadays, numerous studies have shown that PI3K-Akt and MAPK pathways are two of the most frequently mutated oncogenic pathways in cancer and are involved in cancer development [44–46].

Subsequently, we screened the differential genes in HNSCC by dividing the median value of CERCAM expression into two sample groups, high and low. We then performed GSEA enrichment analysis to further identify the pathways associated with CERCAM expression. After screening terms for conditions meeting NOM *P* < 0.05, FDR < 0.25, and |NES|> 1, Fig. 14 c-l shows the top 10 associated terms in the CERCAM high expression group. We found that the CERCAM high expression group was mainly enriched in gene sets related to adhesion and remodeling of cells to the extracellular matrix, including "NABA_CORE_MATRISOME", "REACTOME_EXTRACELLULAR_ MATRIX_ORGANIZATION", "NABA_ECM_GLYCOPROTEINS","REACTOME_DEGRADATION_OF_THE_EXTRACELLULAR_MATRIX","REACTOME_COLLAGEN_FORMATION","KEGG_ECM_RECEPTOR_INTERACTION". Taken together, the results of gene enrichment analysis suggest that high expression of CERCAM may be involved in functional signaling pathways related to cell adhesion in HNSCC to promote cancer development.

## Discussion

As the sixth most common tumor worldwide, head and neck squamous carcinoma (HNSCC) is a major cause of death among cancer patients worldwide each year [47]. Despite the progress in early screening and various types of treatment, the prognosis of HNSCC patients remains disappointing due to its strong metastatic characteristics [48, 49]. Therefore, it is important to urgently explore new biomarkers to study the molecular mechanisms of HNSCC development and to effectively improve patient survival.

CERCAM, as a gene associated with cell adhesion as well as extracellular matrix remodeling, its role in tumors is mainly to promote tumor epithelial cell migration and promote cancer progression [21]. Although it has been found that the expression level of CERCAM in Colon Cancer and kidney cancer affects the prognostic level of tumors [50, 51]. However, in head and neck squamous carcinoma, the prognostic significance and biological functions of CERCAM have never been elucidated. Therefore, this study was based on head and neck squamous carcinoma to investigate the expression pattern, prognostic and diagnostic value, and immunological characteristics of CERCAM, and also to predict the potential pathological mechanisms promoting the progression of head and neck squamous carcinoma.

We analyzed the TCGA-HNSCC dataset and multiple GEO HNSCC datasets, and we first demonstrated that CERCAM mRNA expression levels and total protein expression levels in HNSCC were overexpressed compared to normal tissues, which was consistent with the immunohistochemical staining results of CERCAM in HNSCC in the HPA database. We then analyzed the correlation between CERCAM in head and neck squamous carcinoma and clinicopathological features, and the results showed that the expression of CERCAM correlated with OS, an indicator of survival outcome. It is suggested that CERCAM may correlate with the prognostic level of patients. Therefore, to investigate the prognostic value of CERCAM in HNSCC, we found that the expression level of CERCAM was an independent risk factor for predicting OS, DSS, and PFI by univariate and multivariate COX regression analysis. And further analysis of CERCAM expression levels in clinicopathological subgroups of patients showed that advanced T3 and T4 patients, stage N2 and N3 patients with lymph node involvement, M0 patients without distant metastases, and histologically graded G3 and G4 patients had high CERCAM expression levels associated with poor prognostic indicators. And we also found a satisfactory diagnostic value of CERCAM in HNSCC (AUC: 0.893). However, we found that CERCAM expression levels predicted 3-, 7-, and 10-year survival with suboptimal sensitivity. We hypothesize that the possible reasons for this are that time is a large confounding factor, that the expression of CERCAM in HNSCC has changed over time, thus decreasing the diagnostic efficacy, and that the diagnostic efficacy is also influenced by a variety of factors, and that further research on this is needed in the future. Taken together, these results suggest that CERCAM expression may be associated with increased tumor malignancy and poorer prognosis in HNSCC.

Therefore, we verified the effect of CERCAM on the malignant biology of tumor cells by inhibiting its expression in HNSCC cells in vitro, and the results showed that the downregulation of CERCAM inhibited the viability of HNSCC cells, which was consistent with the previous results. To further investigate the reason for this pro-cancer property of CERCAM, we analyzed the mutation type and methylation level of CERCAM in HNSCC, and the results showed that missense mutation was the main mutation type, while the decrease of methylation level also indicated the pro-cancer property of CERCARM in HNSCC. Based on these results, we have reason to believe that CERCAM functions as an oncogene in HNSCC tumors.

We then analyzed the role of CERCAM in head and neck squamous carcinoma by single cell level and immune cell infiltration level, and we found that CERCAM was expressed on cells including tumor cells and correlated with the infiltration of M2 macrophages in the tumor microenvironment. It is well known that M2 macrophages are among the most important cells in the tumor microenvironment that promote cancer progression [52, 53]. We hypothesized that CERCAM may induce macrophage M2 polarization immune infiltration in HNSCC and provided evidence of CERCAM-induced macrophage M2 polarization in in vitro cellular assays. Finally, we used gene co-expression analysis and gene enrichment analysis to explore the functions of CERCAM in head and neck squamous carcinoma involved in cell adhesion and extracellular matrix remodeling to promote tumor development, as well as predicting the potential oncogenic signaling pathways PI3K-AKT, MAPK, in which CERCAM is involved. In conclusion, this study comprehensively analyzed the expression pattern and prognosis of CERCAM in HNSCC diagnostic value. Based on the preliminary results of this study, CERCAM may serve as an important biomarker and therapeutic target for patients with HNSCC.

However, there are some limitations to our study. Our results are mainly based on data from the Human Cancer Information Public Database. Further in vivo and in vitro experiments are needed to validate the mechanism of action of CERCAM in HNSCC.

## Conclusion

In this study, we demonstrated the aberrant expression pattern of CERCAM in HNSCC with prognostic and diagnostic value. And we provided in vitro evidence that CERCAM promotes malignant biological behavior of tumors and induces macrophage M2 polarization immune infiltration to accelerate their malignant progression. We highlight the clinical significance of CEACAM expression in HNSCC and provide a theoretical basis for further studies on CERCAM-targeted inhibitors for adjuvant treatment of head and neck squamous carcinoma.

### Supplementary Information

**Additional file 1: Table.S1.** The full names of tumor abbreviation from TCGA. **Table.S2.** si-CERCAM sequence. **Table.S3.** qRT-PCR PRIME Sequence. **Table.S4.** Details on TOP30 GSEA for HNSCC patients in the CERCAM high expression group.

## Data Availability

All raw data information analyzed in this study can be found and accessed in the human TCGA (https://portal.gdc.cancer.gov/) public database for all readers to review and analyze. The data on all analysis platforms and websites applied in this study are open and shared, and all readers can obtain the raw data directly or after registering for a free account without payment. All data generated or analyzed in this study can be found in this published article [and its supplementary information file].

## Abbreviations

CERCAM: Cerebral endothelial cell adhesion molecule
HNSCC: Head neck squamous cell carcinoma
ECM: Extracellular matrix
TME: Tumor microenvironment
TAMs: Tumor-associated macrophages
