# Supplementary material for: CERCAM is a prognostic biomarker associated with immune infiltration of macrophage M2 polarization in head and neck squamous carcinoma
Source: BMC Oral Health. 2023 Oct 7;23:724. doi: 10.1186/s12903-023-03421-0 (PMC10559510; doi:10.1186/s12903-023-03421-0)
Supplement: Supplementary file 1 — Additional file 1: Table.S1. The full names of tumor abbreviation from TCGA. Table.S2. si-CERCAM sequence. Table.S3. qRT-PCR PRIME Sequence. Table.S4. Details on TOP30 GSEA for HNSCC patients in the CERCAM high expression group. [file 12903_2023_3421_MOESM1_ESM.docx]

**Table of contents**

1. The full names of tumor abbreviation from TCGA (Table.S1)

2. si-CERCAM Sequence (Table.S2)

3.qRT-PCR PRIME Sequence (Table.S3)

4. Details on TOP30 GSEA for HNSCC patients in the CERCAM high expression group (Table.S4)

Table.S1 The full names of tumor abbreviation from TCGA

| **Abbreviations Tumor full manes** |
| --- |
| ACC Adrenocortical carcinoma |
| BLCA Bladder Urothelial Carcinoma |
| BRCA Breast invasive carcinoma |
| CESC Cervical squamous cell carcinoma and endocervical adenocarcinoma |
| CHOL Cholangiocarcinoma |
| COAD Colon adenocarcinoma |
| COADREAD Colon adenocarcinoma/Rectum adenocarcinoma Esophageal carcinoma |
| DLBC Lymphoid Neoplasm Diffuse Large B-cell Lymphoma |
| ESCA Esophageal carcinoma |
| FPPP FFPE Pilot Phase II |
| GBM Glioblastoma multiforme |
| GBMLGG Glioma |
| HNSC Head and Neck squamous cell carcinoma |
| KICH Kidney Chromophobe |
| KIPAN Pan-kidney cohort (KICH+KIRC+KIRP) |
| KIRC Kidney renal clear cell carcinoma |
| KIRP Kidney renal papillary cell carcinoma |
| LAML Acute Myeloid Leukemia |
| LGG Brain Lower Grade Glioma |
| LIHC Liver hepatocellular carcinoma |
| LUAD Lung adenocarcinoma |
| LUSC Lung squamous cell carcinoma |
| MESO Mesothelioma |
| OV Ovarian serous cystadenocarcinoma |
| PAAD Pancreatic adenocarcinoma |
| PCPG Pheochromocytoma and Paraganglioma |
| PRAD Prostate adenocarcinoma |
| READ Rectum adenocarcinoma |
| SARC Sarcoma |
| STAD Stomach adenocarcinoma |
| SKCM Skin Cutaneous Melanoma |
| STES Stomach and Esophageal carcinoma |
| TGCT Testicular Germ Cell Tumors |
| THCA Thyroid carcinoma |
| THYM Thymoma |
| UCEC Uterine Corpus Endometrial Carcinoma |
| UCS Uterine Carcinosarcoma |
| UVM Uveal Melanoma |
| OS Osteosarcoma |
| ALL Acute Lymphoblastic Leukemia |
| NB Neuroblastoma |
| WT High-Risk Wilms Tumor |

Table.S2 si-CERCAM sequence

| **si RNA** | **Sequence** |
| --- | --- |
| Si-CERCAM#1 | CAGGTTCTACCCAGATGAAGAGG |
| Si-CERCAM#2 | CACCGTTATGGGTACATGAATGT |
| Si-CERCAM#3 | CAGCAAGATAGGGTTTGACGAGG |

Table.S3 qRT-PCR PRIME Sequence

| Gene | Forward (5’-3’) | Reverse (5’-3’) |
| --- | --- | --- |
| GAPDH | CACCCACTCCTCCACCTTTGAC | GTCCACCACCCTGTTGCTGTAG |
| CERCAM | CATGCTCGCCTCGCTCTGG | TCCTGATGGCACTGCTGTTGAG |
| CD68 | CTACTGGCAGAGAGCACTGGAAC | CCTGTGGTGGTTGTCCTGTGAG |
| CD163 | AAAGAATCCCGCATTTGGCAGTG | CAGATAACTCCCGCATCCTCCTTG |
| VSIG4 | TGCCATCATCCTCATCATCTCCTTG | CTGCTTCGTAGACATGCTCTTGTTG |
| CD206 | ACCTCACAAGTATCCACACCATCG | GGGTCCCATCACTCCACTCAAAG |

Table.S4 Details on TOP30 GSEA for HNSCC patients in the CERCAM high expression group

| ID | enrichmentScore | NES | P value | Q value |
| --- | --- | --- | --- | --- |
| NABA_CORE_MATRISOME | 0.749098 | 2.947565 | 1E-10 | 7.45E-09 |
| REACTOME_EXTRACELLULAR_MATRIX_ORGANIZATION | 0.734008 | 2.929491 | 1E-10 | 7.45E-09 |
| NABA_ECM_GLYCOPROTEINS | 0.744237 | 2.824904 | 1E-10 | 7.45E-09 |
| REACTOME_DEGRADATION_OF_THE_EXTRACELLULAR_MATRIX | 0.729053 | 2.706479 | 1E-10 | 7.45E-09 |
| REACTOME_COLLAGEN_FORMATION | 0.788901 | 2.699815 | 1E-10 | 7.45E-09 |
| KEGG_ECM_RECEPTOR_INTERACTION | 0.794928 | 2.688659 | 1E-10 | 7.45E-09 |
| REACTOME_ECM_PROTEOGLYCANS | 0.794707 | 2.645344 | 1E-10 | 7.45E-09 |
| PID_INTEGRIN1_PATHWAY | 0.817563 | 2.6449 | 1E-10 | 7.45E-09 |
| REACTOME_NON_INTEGRIN_MEMBRANE_ECM_INTERACTIONS | 0.812729 | 2.599824 | 1E-10 | 7.45E-09 |
| KEGG_FOCAL_ADHESION | 0.681487 | 2.595681 | 1E-10 | 7.45E-09 |
| REACTOME_ASSEMBLY_OF_COLLAGEN_FIBRILS_AND_OTHER_MULTIMERIC_STRUCTURES | 0.798018 | 2.568702 | 1E-10 | 7.45E-09 |
| REACTOME_COLLAGEN_BIOSYNTHESIS_AND_MODIFYING_ENZYMES | 0.787582 | 2.547381 | 1E-10 | 7.45E-09 |
| REACTOME_INTEGRIN_CELL_SURFACE_INTERACTIONS | 0.735988 | 2.502598 | 1E-10 | 7.45E-09 |
| REACTOME_REGULATION_OF_INSULIN_LIKE_GROWTH_FACTOR_IGF_TRANSPORT_AND_UPTAKE_BY_INSULIN_LIKE_GROWTH_FACTOR_BINDING_PROTEINS_IGFBPS | 0.685063 | 2.498486 | 1E-10 | 7.45E-09 |
| REACTOME_COLLAGEN_DEGRADATION | 0.772979 | 2.494928 | 1E-10 | 7.45E-09 |
| REACTOME_ELASTIC_FIBRE_FORMATION | 0.817654 | 2.468692 | 1E-10 | 7.45E-09 |
| REACTOME_MET_PROMOTES_CELL_MOTILITY | 0.82152 | 2.442204 | 1E-10 | 7.45E-09 |
| PID_AVB3_INTEGRIN_PATHWAY | 0.737193 | 2.436884 | 1E-10 | 7.45E-09 |
| PID_INTEGRIN3_PATHWAY | 0.807386 | 2.413196 | 1E-10 | 7.45E-09 |
| REACTOME_MET_ACTIVATES_PTK2_SIGNALING | 0.864406 | 2.409378 | 1E-10 | 7.45E-09 |
| WP_FOCAL_ADHESION | 0.632879 | 2.407615 | 1E-10 | 7.45E-09 |
| REACTOME_O_GLYCOSYLATION_OF_TSR_DOMAIN_CONTAINING_PROTEINS | 0.816985 | 2.386229 | 1.08E-10 | 7.65E-09 |
| WP_MIRNA_TARGETS_IN_ECM_AND_MEMBRANE_RECEPTORS | 0.9159 | 2.365561 | 1E-10 | 7.45E-09 |
| REACTOME_MOLECULES_ASSOCIATED_WITH_ELASTIC_FIBRES | 0.802739 | 2.337865 | 5.43E-10 | 3.14E-08 |
| WP_ENDOCHONDRAL_OSSIFICATION | 0.727983 | 2.335896 | 1.17E-10 | 7.65E-09 |
| WP_ENDOCHONDRAL_OSSIFICATION_WITH_SKELETAL_DYSPLASIAS | 0.727983 | 2.335896 | 1.17E-10 | 7.65E-09 |
| NABA_COLLAGENS | 0.766747 | 2.314992 | 2.2E-09 | 1.07E-07 |
| REACTOME_COLLAGEN_CHAIN_TRIMERIZATION | 0.766747 | 2.314992 | 2.2E-09 | 1.07E-07 |
| PID_SYNDECAN_1_PATHWAY | 0.766906 | 2.31482 | 3.77E-10 | 2.25E-08 |
| REACTOME_SIGNALING_BY_MET | 0.69258 | 2.31348 | 1E-10 | 7.45E-09 |
